# How professional training impacts teaching innovation among ideological and political teachers: the mediating and moderating role of basic psychological needs satisfaction

**DOI:** 10.3389/fpsyg.2023.1246951

**Published:** 2023-11-03

**Authors:** Can Cui, Yanjun Yin

**Affiliations:** ^1^School of Marxism, China University of Petroleum-Beijing, Beijing, China; ^2^Department of Education, Ocean University of China, Qingdao, China

**Keywords:** professional training, basic psychological needs, teaching innovation, ideological and political teachers, mediating effect, moderating effect

## Abstract

The aim of this study is to explore the impact of professional training on teaching innovation among senior high school ideological and political teachers. By introducing the concept of basic psychological needs satisfaction as a mediating factor, the study seeks to uncover the mechanisms and patterns that manifest in teaching innovation within the Chinese cultural context. To gather empirical data, a questionnaire survey was administered to a sample of 780 teachers in this specialized field. The results showed that providing more professional training is an effective way to enhance teaching innovation. Drawing on Self-Determination Theory, the satisfaction of basic psychological needs (competence, relatedness, and autonomy) was found to play an important role in this process. Competence need satisfaction and relatedness need satisfaction played a mediating role, while autonomy need satisfaction played a moderating role. Based on these findings, several recommendations are provided to support the professional training of senior high school ideological and political teachers and enhance their innovation, including providing personalized training programs, building a mentor system, and allowing greater autonomy in school management.

## 1. Introduction

Innovation is not a new phenomenon (Fagerberg, 2006). As societal progress and technological advancements continue to unfold, the imperative for innovation grows increasingly salient. For a corporation, innovation is considered to be the corner stone of the success and survival (Yesil and Sozbilir, 2013). In schools, innovation also plays a pivotal role. Creative atmosphere “will change a classroom from a four walled room filled with educational hopes into an environment that is infused with excitement, curiosity, and genuine student learning” (Simplicio, 2000). In the classroom, the teacher’s innovative behavior and creative teaching strategies (Mahajan and Kaushal, 2017) can enhance students’ creativity. A classroom environment imbued with a culture of innovation can significantly enhance students’ academic engagement and interest (Khikmah, 2019), academic record will also improve accordingly (Baghaei and Riasati, 2015). Since the onset of the COVID-19 pandemic in 2019, measures such as restrictions on movement and large gatherings have been implemented in order to mitigate the risk of infection. As a result, teachers and students have had to adapt and find new ways to continue teaching and learning remotely (Bozkurt et al., 2020). One such approach, known as blended learning, combines traditional face-to-face and online components. While such changes may bring about overall positive effects for teachers, they can also introduce complications as the specifics of the change process are often uncertain. To address this uncertainty and maintain teaching efficiency, teachers must seek out new approaches in their daily work.

In China, beginning in 2019, the government has successively issued a series of directives, such as “Opinions on Deepening the Reform and Innovation of Ideological and Political Theory Courses in Schools for the New Era” and “Implementation Plan for the Reform and Innovation of Ideological and Political Theory Courses in Schools for the New Era.” These directives aim to orchestrate the integrated development of the “Integrated Ideological and Political Course in Schools and Universities” across primary, secondary, and tertiary education levels. Within this integrated framework, senior high school-level courses serve as a critical juncture, bridging the foundational and advanced aspects of this education model. In China, senior high school ideological and political courses are predicated on students’ cognitive development and focus on enhancing their political literacy. These courses are comprehensive in scope, encompassing not only knowledge-based requirements but also extending to diverse dimensions such as values, ethics, and psychological well-being. The multifaceted nature of the content inherently demands higher levels of teaching innovation. Teaching approaches are increasingly shaped by the influence of internet and digital technologies (Henderson et al., 2017; Parong and Mayer, 2018). Regardless of whether this impact is positive (Lin et al., 2020) or negative (Falck et al., 2018), it sets forth new imperatives for teaching innovation in senior high school ideological and political courses. Furthermore, the unique developmental characteristics of senior high school students can impact the effectiveness of teaching and the innovation in instructional methods to a certain degree (Tong et al., 2003). Therefore, whether guided by policy directives or necessitated by objective developmental imperatives, teaching innovation in ideological and political courses is requisite to adapt to their evolving landscape.

Under such circumstances, educational institutions should offer effective training to teachers (Rasheed et al., 2020). According to previous studies, professional training might have an impact on teaching innovation, but its internal impact mechanism is not clear and needs to be further explored. Scholars have yet to engage in data-driven analyses or empirical investigations specifically targeting the factors that influence teaching innovation in ideological and political courses at the senior high school level in China. This study aimed to improve the teaching ability of Chinese senior high school ideological and political teachers by investigating how professional training impacts their teaching innovation. This focus affords a more comprehensive and objective view of current professional training and offers some individualized approaches for professional training from a psychological perspective. The study concurrently furnishes empirical data rooted in the Chinese context, as well as insights framed within the cultural milieu of the nation.

## 2. Literature review and development of hypotheses

### 2.1. Teaching innovation and influencing factors

In the realm of teachers’ professional development, innovation competence is a cornerstone that covers multifarious facets of a teacher’s responsibilities within the school environment. Teaching innovation is defined as the origination or adoption of novel and efficacious pedagogical practices, methodologies, or educational materials in the classroom setting (Anderson et al., 2014). Innovative teaching is fundamentally predicated upon fostering a culture of creative learning and stimulating proactive student engagement through the integration of emerging ideas, approaches, tools, and subject matter (Kahramonovna, 2021).

The concept of innovation is multifaceted, being perceived either as an outcome, a process, or even a mindset (Kahn, 2018). Some scholars posit that innovation is an amalgamation of ideas, practices, and eventual outcomes, encompassing stages such as problem perception, hypothesis formation and testing, modification, and ultimate resolution (Rerke et al., 2020). Typically, this iterative process is either executed by the educator or collaboratively with peers (Messmann et al., 2022).

To conduct a comprehensive study of teaching innovation, it is imperative to scrutinize the variables that influence it. Numerous studies have demonstrated that innovation is significantly affected by organizational factors such as leadership (Samsir, 2018; Villaluz and Hechanova, 2019), transformational leadership (Messmann et al., 2022), involvement climate (Naqshbandi et al., 2019), teacher training (Artacho et al., 2020), knowledge sharing (Kim and Shim, 2018), organizational learning (Putra et al., 2020), collaborative culture (Yang et al., 2018), and a plethora of institutional factors such as school culture, peer support, and resource availability (Davies et al., 2014; Cai and Tang, 2022).

In addition to organizational variables, personal factors of the educator also play a substantial role in shaping the readiness and creativity levels for introducing pedagogical innovations. These include the educator’s motivation for success, value orientations, propensity for calculated risk-taking, and willingness to assume responsibility in relation to risks and creativity (Rerke et al., 2020). Individual capabilities such as domain-specific expertise, knowledge process (Imran et al., 2018), knowledge search (Wang et al., 2019), and intrinsic motivations for innovation (Rosenblatt, 2011) significantly affect the innovation landscape. A research study conducted on more than 1,770 PhD scientists and engineers at Cambridge University posits that although both formal and informal incentives must be considered, individual motives warrant particular attention (Sauermann and Cohen, 2010). Therefore, scrutinizing the impact of these individualized variables is essential for the advancement of teaching innovation. It is noteworthy that organizational factors ultimately exert their influence on teaching innovation by shaping individual teacher characteristics.

### 2.2. Professional training and innovation

In advancing the discourse on teachers’ professional trajectory, a multi-dimensional approach to teacher training is indispensable. Teacher training encompasses pre-service stages, such as formal education at the university level and practical internships (Fuentes-Abeledo et al., 2020), along with in-service professional development and experiential learning acquired through occupational practices. Interestingly, empirical evidence suggests no significant correlation between pre-service training or examination scores and teacher productivity (Harris and Sass, 2011).

Within the sphere of human resource management, professional training is conceptualized as “planned and systematic activities aimed at enhancing the acquisition of knowledge, skills, and attitudes” (Salas et al., 2012). Contrasted with general education, professional training holds a specific orientation toward skill application, aiming primarily to facilitate the transference of acquired knowledge into the workplace (Bonnes and Hochholdinger, 2020). Transposing this definition to the domain of teacher development, professional training for teachers is orchestrated learning activities designed to foster the elevation and refinement of pedagogical knowledge, competencies, and instructional quality, implemented either socially or institutionally. It can be bifurcated into hard-skill trainings, which include domain-specific knowledge, instructional methodologies, and cutting-edge instructional technologies like digital competence (Artacho et al., 2020), and soft-skill trainings, encompassing creativity, collaborative teaching, professional ethics, and pedagogical attitudes.

The relationship between professional training and innovation is close. From an organizational management standpoint, investment in human capital is crucial for enhancing an enterprise’s innovative capacity (Mariz-Perez et al., 2012). Empirical studies within industrial sectors have demonstrated that well-trained employees act as direct catalysts for innovation (González et al., 2016).

For teachers, both hard and soft skill training are conducive to fostering teaching innovation. Research indicates that specialized competency training, such as digital literacy, plays an instrumental role in encouraging pedagogical innovation (Artacho et al., 2020). Enhancement in teaching abilities through professional development not only augments teachers’ skill sets but also invigorates their creative potential (Harris and Sass, 2011). The advantages of such training encompass knowledge acquisition, the nurturing of collaboration, the cultivation of inventive thought, and the development of problem-solving abilities (Kottke, 1999)—all of which are vital components of teaching innovation. Therefore, we posit the following hypothesis:

*Hypothesis 1 (H1)*: Teacher professional development positively impacts pedagogical innovation.

Further investigation is warranted to explore how specific hard and soft skills contribute to teacher-led innovation. Beyond training related directly to innovation skills, could other forms of professional training also stimulate creative teaching practices? As previously elucidated, motivation serves as a critical catalyst in the innovation equation. Consequently, it becomes imperative to delve into the underlying mechanisms by which teacher professional development influences innovation, particularly emphasizing the pivotal role of motivation.

### 2.3. Satisfaction of basic psychological needs

Self-Determination Theory (SDT) introduces three basic psychological needs: competence, relatedness, and autonomy (Deci et al., 2017). Competence relates to an individual’s sense of control over their environment and the desire to acquire new skills. Relatedness concerns positive relationships and social support, while autonomy pertains to individual control over one’s actions and perceptions of psychological freedom.

According to the SDT, motivation can be categorized into two types: intrinsic and extrinsic (Ryan and Deci, 2000). When examining the motivational factors influencing teacher innovation, extrinsic motivation can manifest both materially, such as monetary rewards, and immaterially, such as recognition (Rosenblatt, 2011). However, some studies suggest that extrinsic rewards may adversely affect the workplace environment by engendering competitive behaviors that stifle relationships, hinder openness and learning, discourage risk-taking, and ultimately undermine intrinsic work motivation (Ahmed, 1998). Conversely, a considerable body of research posits that many innovations are initially fueled by intrinsic motivations (Kumar and Namrata, 2022). For instance, the quest for intellectual challenges has been found to be strongly correlated with both the effort expended and the innovative performance achieved (Rosenblatt, 2011). Teachers often derive satisfaction from solving problems (Stephan, 1996) or experience intellectual excitement when seeing their ideas come to fruition (Davenport et al., 2003). Intrinsic motivation has been shown to have a direct positive impact on individual innovative behavior, whereas extrinsic motivation has an indirect positive impact mediated by intrinsic motivation (Xu et al., 2009). Thus, by studying the mechanisms to transform extrinsic motivation into intrinsic motivation, policies can be more effectively formulated to promote teacher innovation. Ryan and Deci’s research corroborates that satisfying basic psychological needs facilitates this transformation (Ryan and Deci, 2000).

Professional training can enhance the sense of need satisfaction. Once intrinsic motivation is augmented, it can promote teaching innovation. Therefore, the role of the three basic psychological needs in promoting innovation should be considered in this study. Satisfaction of the three basic psychological needs, is typically used as a composite, but sometimes analyzed with each need separately. For example, one study found that transformational leaders could support the satisfaction of these needs in distinct ways (Messmann et al., 2022). Given the prior analysis that professional competence is a key determinant of teaching innovation and that professional training is a pivotal mechanism for skill enhancement which can meet the need of competence need. we formulate the following hypothesis:

*Hypothesis 2 (H2)*: The satisfaction of the competence need (SOCN) mediates the relationship between teachers' professional training and teaching innovation.

For teachers, teaching innovation will become considerably challenging without ample discussion, collaboration, and peer support (Liu et al., 2022). Professional training aims to improve such collaborative abilities which can meet the need of relatedness need., leading to the next hypothesis:

*Hypothesis 3 (H3)*: The satisfaction of the relatedness need (SORN) mediates the relationship between teachers' professional training and teaching innovation.

Previous studies have found that autonomy need satisfaction can have a significant moderating effect in certain situations. For example, a study of 184 New Zealand employees found job autonomy has a significant interaction effect on the relationships between distributive justice and turnover intentions (Haar and Spell, 2009). Autonomy need satisfaction is primarily attributed to the decentralization of management. This factor operates independently of the training and innovation processes, yet it could potentially affect the strength of the relationship between professional training and innovation. In the cultural context of China, teachers may be particularly inclined to defer to authority (Zheng et al., 2021), which could potentially influence teaching innovation. Thus, the study proposes the final hypothesis:

*Hypothesis 4 (H4)*: The satisfaction of the autonomy need (SOAN) moderates the relationship between teachers' professional training and teaching innovation.

## 3. Materials and methods

### 3.1. Sample and data collection procedures

In this study, the data were collected from senior high school ideological and political teachers in the S province of China. We distributed 900 questionnaires using the principle of random stratified sampling and received 825 responses, yielding a recovery rate of 91.6%. Out of these responses, 780 were found to be valid after removing questionnaires with excessive repetition or missing values (54 questionnaires removed), resulting in an effective rate of 94.5%. A descriptive analysis was performed on the sample by gender, age, teaching experience, professional title, and the type of school. The results are presented in Table 1.

**Table 1:** Demographics statistics.

Variable	Categories	Number of samples	Proportion
Gender	Male	296	37.9%
	Female	484	62.1%
Age	25 and under	44	5.6%
	26–35	218	27.9%
	36–45	340	43.6%
	46—50	106	13.6%
	51 and above	72	9.2%
Teaching experience	Less than 3 years	100	12.8%
	3–5 years (inclusive)	75	9.6%
	5–10 years (inclusive)	86	11.0%
	10 or more years	519	66.5%
Title	Top tier	3	0.4%
	Senior	139	17.8%
	Intermediate (or Level 1)	339	43.5%
	Junior (or Levels 2 and 3)	257	32.9%
	Unclassified	42	5.4%
School type	Public	675	87%
	Private	105	14%

### 3.2. Measures

In this study, three measurement tools were utilized: teachers’ professional training scale, the satisfaction of basic psychological needs scale, and the teaching innovation scale. The satisfaction of basic psychological needs scale is comprised of three dimensions: competence need satisfaction scale, relatedness need satisfaction scale, and autonomy need satisfaction scale. These dimensions were measured separately and analyzed individually. All scales utilized a 5-point Likert scoring system, with responses ranging from 1 (strongly disagree) to 5 (strongly agree). The questionnaire items were revised following a small-scale test and the reliability and validity of the revised questionnaire were tested. The validity of the measurement tools was assessed using confirmatory factor analysis.

#### 3.2.1. Teachers’ professional training scale

In the study, the professional training scale changes from the Extensive Training Scales (Sun et al., 2007), which contains 5 items (a sample item was, “Our institution is willing to invest substantial amounts of time and financial resources in the professional development of our teaching staff”). The internal consistency of the measure was determined using SPSS, resulting in an alpha coefficient of 0.929, indicating a high degree of internal consistency. Additionally, the construct reliability (CR) was calculated to be 0.9318 and the average variance extracted (AVE) was determined to be 0.7335, providing further evidence of the measure’s reliability.

#### 3.2.2. Satisfaction of basic psychological needs scale

The basic psychological needs satisfaction scale comes from the research results of Deci et al. (2001). It consists of three subscales:

The SOCN contains six items (Cronbach’s α = 0.773; a sample item was, “I frequently have the opportunity to acquire new skills that I am interested in during my work.”).

The SORN contains eight items (Cronbach’s α = 0.878; a sample item was, “I have a good relationship with my colleagues.”)

The SORN contains seven items (Cronbach’s α = 0.781; a sample item was, “I have the flexibility to decide on my operation way of working according to my preferences.”)

#### 3.2.3. Teaching innovation scale

The scale of teachers’ teaching innovation in this study comes from an empirical study of teachers’ professional learning (Liu et al., 2016), with a total of 5 items.

The first two items measure teachers’ innovative thinking (sample item, “In my teaching practice, I prefer to adopt a creative approach by applying various teaching techniques, methodologies, or teaching philosophies that promote the engagement of students’ cognitive processes.”).

The third is the communication of innovative thinking with colleagues (“I enjoy engaging in communication and sharing my new ideas with colleagues, in order to gain support and recognition.”).

The last two items measure the competence to implement innovation (sample item, “In order to realize my new ideas in teaching, I will strive to acquire the necessary resources.”). The five items comprehensively represent teachers’ teaching innovation. The calculated alpha value was 0.935 with good reliability. The CR value was 0.9318, AVE 0.75, and the validity was acceptable.

### 3.3. Data analysis

Before carrying out hypothesis testing, the discriminant validity among the multi-item constructs and common method variance were assessed to ensure the validity of the measurement model. Pearson’s correlation analyses were carried out to lay a foundation for hypothesis testing. The hypotheses were tested using structural equation modeling. The analytic strategy proceeded in three stages. (1) The main descriptive statistics, correlation matrix, differences, and stratified regression analysis were computed using SPSS. (2) Structural equation modeling (SEM) was used to explore the mediating effects using the software AMOS. SEM was selected for this study because it allows for the examination of complex relationships among variables in a more comprehensive manner. (3) The moderated effect was tested using the PROCESS macro for SPSS (Hayes, 2017). All SEM-based tests in this study were conducted through a bootstrapping procedure with a bootstrap sample of 5,000.

## 4. Result

### 4.1. Descriptive statistics and correlation matrix

The descriptive statistics and correlation matrix of each main variable are presented in Table 2.

**Table 2:** Variable description statistics and correlation matrix.

	Mean	Standard deviation	1	2	3	4
1. Autonomy need satisfaction	3.563	0.707	1			
2. Competence need satisfaction	4.112	0.621	0.589**	1		
3. Relatedness need satisfaction	4.357	0.584	0.505**	0.752**	1	
4. Professional training	4.234	0.834	0.465**	0.447**	0.454**	1
5. Teaching innovation	4.399	0.643	0.474**	0.661**	0.610**	0.518**

As demonstrated in Table 2, the average values of all research variables are largely between 3.5 and 4.4, indicating that the basic needs of the sample teachers are largely satisfied. Among these, relatedness need satisfaction (*M* = 4.357, SD = 0.584) and competence need satisfaction (*M* = 4.112, SD = 0.621) are particularly well-satisfied. However, the degree of satisfaction with regard to autonomy need satisfaction (*M* = 3.563, SD = 0.707) was slightly lower. Teachers’ perception of professional training (*M* = 4.234, SD = 0.834) was high, as was their level of teaching innovation (*M* = 4.399, SD = 0.643). The correlation between all the research variables reached the significance level of 0.01 and above, indicating a significant positive correlation. Analysis of the correlation matrix reveals that the correlations between variables are consistent with theoretical expectations and that the mutual prediction relationships between variables can be further explored and discussed in the context of the model.

### 4.2. Analysis of differences among groups

The results of a one-way analysis of variance showed a positive correlation between teaching experience and teaching innovation. Specifically, the data indicated that teachers with more than 10 years of experience had significantly higher levels of teaching innovation compared to those with less than 3 years and 3–5 years of experience.

In China, public schools are established and run by the government, while private schools are established and run by non-governmental organizations such as enterprises. The results of the comparison between professional training and teaching innovation among public and private senior high school teachers showed that teachers in public schools had significantly higher levels of both teaching innovation (*M* = 4.431 for public vs. *M* = 4.191 for private) and professional training (*M* = 4.269 for public vs. *M* = 4.008 for private) compared to those in private schools.

### 4.3. Stratified regression analysis

A hierarchical regression analysis was conducted to examine the ability of professional training and psychological needs to predict teaching innovation. The tolerance of all factors was found to be above 0.5, and the variance inflation factor (VIF) was less than 2, indicating the absence of serious collinearity issues among the factors. Control variables, including gender, teaching experience, and school type, were included in the regression model. The goodness-of-fit R-squared of the control variables and teaching innovation was 0.024, indicating that they explained a small portion of the variance. However, the overall *F* value of the multiple regression test was 7.368, reaching statistical significance at the 0.001 level. These results suggest that there is a significant positive relationship between teaching experience and teaching innovation (Table 3).

**Table 3:** Stratified regression analysis results.

Variable		Dependent variable		
		Model 1	Model 2	Model 3
Control variables	Gender	−0.018	−0.034	−0.033
	Teaching experience	0.111**	0.1**	0.076**
	School type	−0.086*	−0.036	0.001
Independent variable	Professional training		0.51***	0.237***
Intermediate variable	Autonomy need satisfaction			0.058
	Competence need satisfaction			0.361***
	Relatedness need satisfaction			0.199***
Regression model summary	*R*^2^	0.024	0.281	0.518
	*F*	7.368***	77.03***	120.449***

*Indicates significance at the 0.05 level.

**Indicates significance at the 0.01 level.

***Indicates significance at the 0.001 level.

Next, professional training was added to the regression model. The goodness-of-fit R-squared for model 2, which included the autonomy variables, was 0.281, indicating that it explained 28.1% of the variance in teaching innovation. The *F* value for significant change was 77.03, reaching statistical significance at the 0.001 level, indicating a good overall fit of the equation. The standard coefficient β for professional training was 0.51, which was statistically significant at the 0.001 level. These results demonstrate that professional training has a significant positive effect on teaching innovation.

Finally, the three dimensions of basic psychological needs were added to the regression model. The goodness-of-fit R-squared for model 3, including the autonomy variables, was 0.518, an increase of 23.7% compared to model 2. The *F*-value for significance change was 120.449, reaching statistical significance at the 0.001 level. The standard coefficient β shows that the SOCN and relatedness need has a significant positive effect on teaching innovation. However, the SOAN did not reach statistical significance, with the lowest coefficient of 0.058. It can also be found that the regression coefficient of professional training is reduced to 0.237 after adding the variable of psychological needs, which is still significant. Further tests for the mediating and moderating effects can be conducted.

### 4.4. The mediating effect of competence need satisfaction

**Figure 1:**
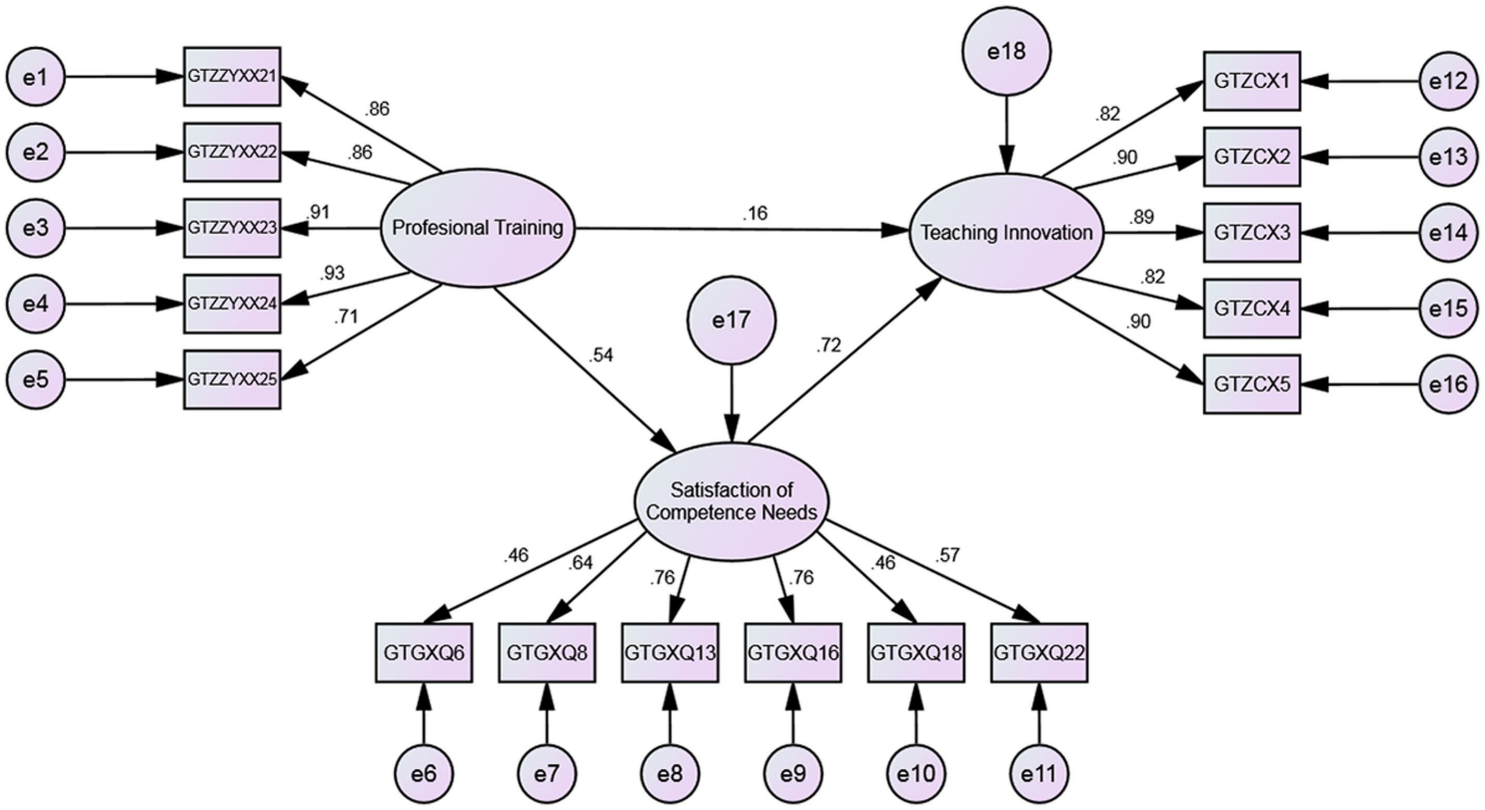
Mediating effects of competence need satisfaction in structural equation model.

The structural equation model is a statistical tool used to evaluate relationships between variables. In this study, it was used to investigate the relationship between competence need satisfaction, professional training, and teaching innovation. The results of the model are depicted in Figure 1.

The fit of the mediating model of competence need satisfaction was analyzed. The key fit indices *X*^2^/df = 2.852, GFI = 0.91, TLI = 0.934, CFI = 0.944, RMSEA = 0.08. The results indicate that the GFI, TLI, and CFI values in the above indices are all greater than 0.9, the chi-square to degrees of freedom ratio is under 3, and the RMSEA value is equal to 0.08, indicating a good fit.

The Bootstrap method was employed to estimate the mediation model using Amos software. The results of the data analysis show that all path coefficients are significant, indicating that competence need satisfaction acts as a substantial mediating factor in the relationship between professional training and teaching innovation.

The total effect of professional training on teaching innovation is the sum of all the path coefficients, while the product of the path coefficients from professional training to competence need satisfaction and then to teaching innovation is the mediating effect. The calculation of the mediation and total effects was performed based on the obtained path coefficients as follows: the indirect effect was calculated to be 0.383 (0.535 * 0.715), and the total effect was calculated to be 0.543 (0.16 + 0.383). The proportion of the indirect effect to the total effect was 70.53%, which exceeds the direct effect of professional training. The results indicate a strong mediating effect of competence need satisfaction on the relationship between professional training and teaching innovation.

### 4.5. The mediating effect of relatedness need satisfaction

**Figure 2:**
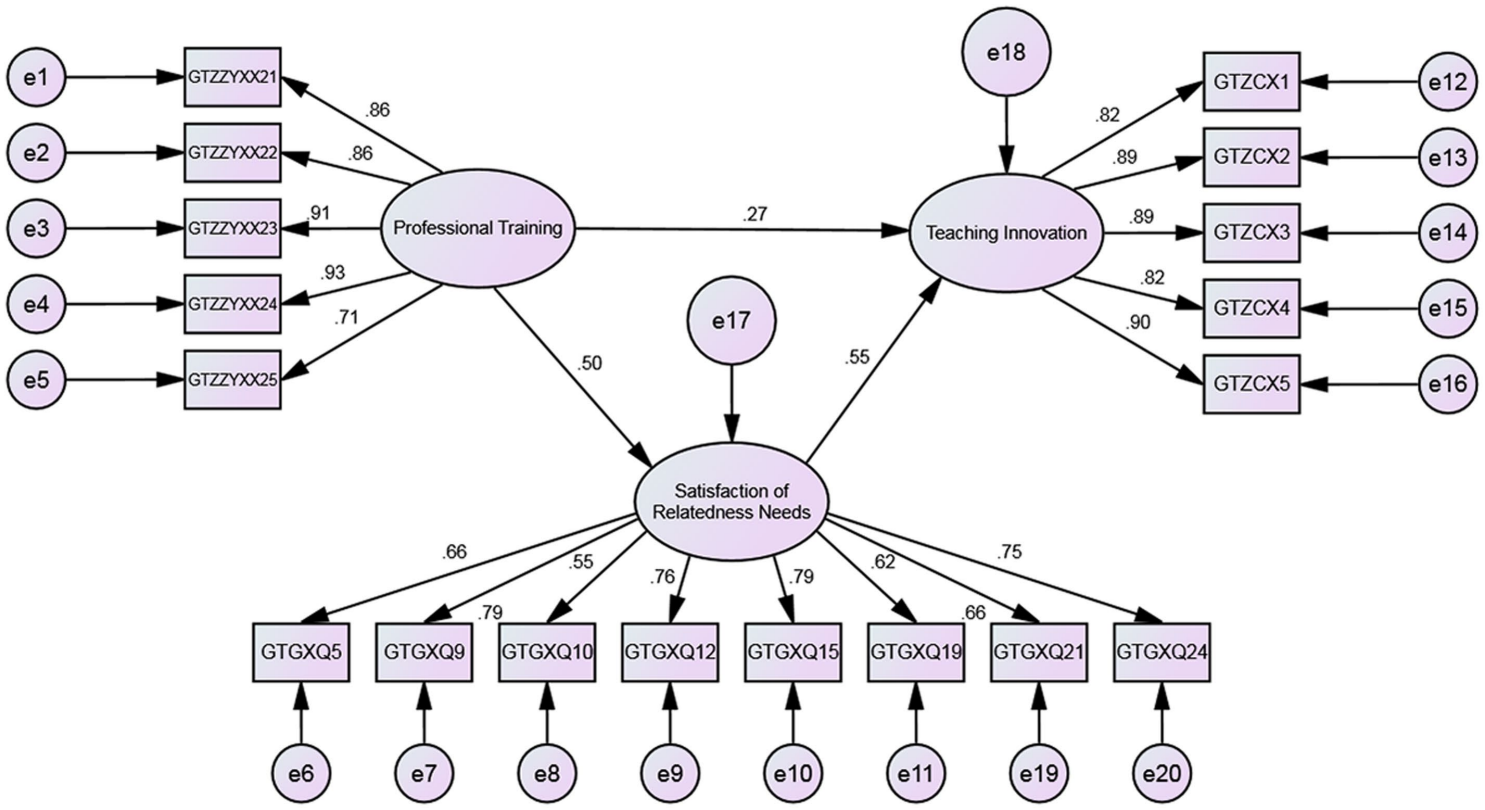
Mediating effects of relatedness need satisfaction in structural equation model.

To further examine the relationship between relatedness need satisfaction, professional training, and teaching innovation, a structural equation model was constructed and tested using Amos software. The results of the model are depicted in Figure 2.

The fit of the mediating model of relatedness need satisfaction was analyzed. The key fit indices *X*^2^/df = 2.915, GFI = 0.941, TLI = 0.928, CFI = 0.938, RMSEA = 0.08. The results indicate that the GFI, TLI, and CFI values in the above indices are all greater than 0.9, the RMSEA value is equal to 0.08, and the chi-square to degrees of freedom ratio is under 3, indicating a good fit.

The Bootstrap method was employed to estimate the mediation model using Amos software. The data reveals that each path coefficient is significant, indicating that relatedness need satisfaction acts as a substantial mediating factor in the relationship between professional training and teaching innovation.

The calculation of the mediation and total effects was performed based on the obtained path coefficients as follows: the indirect effect was calculated to be 0.279 (0.535*0.715), and the total effect was calculated to be 0.544 (0.265 + 0.279). The proportion of the indirect effect to the total effect was 51.29%, which slightly exceeds the direct effect of professional training. The results indicate a significant mediating effect of relatedness need satisfaction on the relationship between professional training and teaching innovation.

### 4.6. Moderating effect of autonomy need satisfaction

The moderating effect of autonomy need satisfaction on the relationship between professional training and teaching innovation was analyzed using Model 1 in Process (Hayes, 2017), a plug-in of SPSS. To prevent collinearity between interaction terms and other factors, it is necessary to decentralize the professional training variable and the autonomy need satisfaction variable.

The adjusted R squared value is 0.354, indicating that this model explains 35.4% of the variance in teaching innovation, which is significant at the 0.001 level. The regression model of teaching innovation reveals that the *p* value of the interaction term between professional training and autonomy need satisfaction is significant at a level lower than 0.001, indicating a significant moderating effect. The R-square value of the interaction term is 0.0163, indicating an increase in the model’s explanatory power for variance in teaching innovation by 1.63%. When autonomy need satisfaction is high (M + 1SD = 0.7065), the simple slope is 0.4251 and is significant. Similarly, when autonomy need satisfaction is low (M − 1SD = −0.7065), the simple slope is 0.2473 and is also significant. This demonstrates that professional training has a greater positive impact on teaching innovation when teachers feel high autonomy need satisfaction. However, when autonomy need satisfaction is low to a certain extent (less than 1.7853), the impact becomes insignificant. These findings suggest that autonomy need satisfaction plays a notable moderating role in the relationship between professional training and teaching innovation.

## 5. Discussion

The results of this study suggest that teachers with more years of experience tend to have higher levels of teaching innovation compared to those with less experience. However, prior research has yielded conflicting conclusions on this topic, with some studies indicating a negative relationship between teaching experience and innovation (Ghaith and Yaghi, 1997). The discrepancy in conclusions may be due to differences in the innovation environment of different organizations. In schools with a positive innovation atmosphere, experienced teachers tend to have a wealth of innovative thinking, leading to higher levels of innovation. In contrast, in schools with a negative innovation atmosphere, experienced teachers may have a lower level of innovation due to a lack of enthusiasm for new ideas. This study emphasizes the significance of exploring the underlying reasons behind the impact of professional training on teaching innovation, as superficial factors may not reveal the underlying mechanisms.

The data indicate a significant positive relationship between professional training and teaching innovation. Hypothesis 1 is verified. This finding is consistent with previous research in related fields, such as a study of 5,200 Norwegian enterprises which found a positive relationship between enterprises’ use of employee training and their innovation activities (Børing, 2017), a Canadian workplace and employee survey which found that more training leads to more product and process innovation (Dostie, 2018). Another study of a Canadian energy company found that training for creativity and innovation can develop individual creative skills for exploration (Rampa and Agogué, 2021). This conclusion has important implications for teachers’ professional development programs, as it highlights the role of professional training in promoting innovative teaching practices.

The data also indicate that the SOCN mediates the relationship between professional training and teaching innovation among senior high school ideological and political teachers. Hypothesis 2 is verified. Firstly, for teachers, professional training can provide the professional knowledge, teaching methods, and information technology required for teaching. The increase of knowledge can satisfy the competence need. This finding aligns with previous research on employees, which found that training is an important factor in the knowledge absorption process and can satisfy the competence need (Neirotti and Paolucci, 2013). Secondly, training has also been shown to encourage openness to new ideas, which can be a source of technological and organizational innovations (Neirotti and Paolucci, 2013). Research has shown that satisfying teachers’ competence need can increase their confidence levels, which in turn has a positive correlation with entrepreneurial activity (Ebert et al., 2019). The acquisition of training knowledge involves the external internalization of knowledge. However, when the training content is more targeted to meet teachers’ competence need, this external internalization process transforms into integration. This internal drive motivates teachers to apply knowledge more creatively during the teaching process.

Moreover, the data shows that the SORN mediates the relationship between professional training and teaching innovation. Hypothesis 3 is verified. Professional training has the potential to enhance teachers’ satisfaction of their relatedness need, then increase their teaching innovation. The investments made in training and development by organizations create an environment allowing for the exchange of knowledge and ideas among employees (Lau and Ngo, 2004). For teachers as well, participation in professional training can also create a positive communication atmosphere. Such communication can increase teachers’ sense of relatedness, as they build trust and engage in more communication and sharing. When teachers feel a high level of trust in their colleague relationships, they are more likely to engage in collaboration. Studies in the manufacturing industry have shown that the higher the knowledge sharing, the higher the innovation behavior of members (Hyoseop and Hongin, 2020). When teachers’ relatedness need is satisfied, they are more likely to engage in collaboration and communication with their peers, which can foster the sharing and refinement of ideas and ultimately generate more innovative ideas. Additionally, teachers are more inclined to proactively seek additional resources and support from colleagues to implement innovative teaching practices, thereby facilitating the adoption of innovative behaviors. By providing professional training for teachers, schools can create a more positive and supportive environment, promoting not only teachers’ professional growth but also their teaching innovation.

Finally, the results support Hypothesis 4, as the SOAN moderates the relationship between professional training and teaching innovation. One possible explanation is that when the need for autonomy is satisfied, people feel more interested, engaged, and happy (Niemiec and Ryan, 2013). When teachers experience greater autonomy, they are more likely to engage in active and open-minded thinking during the training process, which may lead to the generation of more innovative ideas. So that satisfying the autonomy need of teachers could lead to a higher impact of professional training on teaching innovation. This study is also in line with previous research indicating that job autonomy has a significant effect on innovation behavior (Hyoseop and Hongin, 2020) and that granting individuals and teams more freedom promotes innovation (Lumpkin and Dess, 1996). However, top-down management pressure reduces teachers’ sense of autonomy and hinders teaching innovation (Cai and Tang, 2022). The data shows that the mean value for autonomy need satisfaction (3.563) was lower than the levels of competence need satisfaction (4.112) and relatedness need satisfaction (4.357) surveyed in China. These findings suggest that teachers in China have a significant need for autonomy that is not being adequately satisfied. Based on this, it can be inferred that a higher need for autonomy satisfaction among the sampled teachers would lead to a more pronounced moderating effect. Therefore, granting teachers more autonomy is beneficial.

## 6. Practical implications and limitations

Emphasize the importance of senior high school ideological and political teachers’ professional training and create a mentor system (Hobson et al., 2009) where experienced teachers guide beginning teachers. This will help beginning teachers improve their skills quickly, handle basic tasks easily, and focus on teaching innovation. The mentor system can benefit both experienced and beginning teachers by combining the beginning teachers’ innovative ideas with the experienced teachers’ knowledge and experience, leading to an overall increase in innovation.

Schools should create personalized training programs for teachers at different career stages, taking into account their competence and relatedness needs. To meet competence need, professional training should align with teachers’ personal development goals. To meet relatedness need, schools should encourage teachers to participate in professional training together. Grant teachers’ greater autonomy need satisfaction in school management to boost their motivation and encourage innovation. By giving teachers more control over the professional training process and allowing them to experiment with new methods, schools can create an open, inclusive, and innovative environment that promotes teachers’ professional development. It is suggested that schools should allocate resources and offer support to facilitate teachers’ SOAN and encourage the exploration of innovative teaching approaches.

This study also has limitations. The S Province selected for this survey is a region with moderate economic development that offers a conducive economic and external exchange environment. The overall level of school-provided professional training for teachers in this region is higher than the national average. However, the conclusion from this study may not be effective in a region with a lower economy. In the future, it may be advisable to expand the sampling scope and analyze differences, such as differences in economic development, geographic location, and other factors.

It is also worth considering whether the findings of this study, which focused on senior high school ideological and political teachers, can be generalized to teachers at the primary and secondary education levels, or even to university teachers. Further research could examine the applicability of these conclusions to other contexts.

## Data availability statement

The raw data supporting the conclusions of this article will be made available by the authors, without undue reservation.

## Ethics statement

The studies involving human participants were reviewed and approved by the Ethics Committee of China University of Petroleum. The patients/participants provided their written informed consent to participate in this study.

## Author contributions

All authors listed have made a substantial, direct, and intellectual contribution to the work and approved it for publication. CC led the study design and manuscript writing. YY conducted the data collection, analysis, and interpretation. All authors contributed to manuscript revision, read, and approved the submitted version.
